# NIR-Propelled Biomimetic Nanomotors for Photothermal/Chemodynamic/NO Synergistic Tumor Therapy

**DOI:** 10.34133/cbsystems.0495

**Published:** 2026-04-24

**Authors:** Ming Yang, Jian Hu, Zerui Li, Hanhan Xie, Hongri Gu, Chengzhi Hu

**Affiliations:** ^1^ School of Materials Science and Engineering, Harbin Institute of Technology, Harbin 150001, China.; ^2^ Shenzhen Key Laboratory of Biomimetic Robotics and Intelligent Systems, Department of Mechanical and Energy Engineering, Southern University of Science and Technology, Shenzhen 518055, China.; ^3^ School of Optoelectronic Engineering, Guangdong Polytechnic Normal University, Guangzhou 510665, China.; ^4^ Division of Integrative Systems and Design, Hong Kong University of Science and Technology, Hong Kong, China.

## Abstract

Multimodal nanotherapeutic systems capable of integrating photothermal, catalytic, and gas-mediated strategies offer powerful opportunities to overcome the limitations of single-mode cancer therapies. Here, we develop a near-infrared (NIR) light-activated biomimetic nanomotor for targeted nitric oxide (NO) delivery and synergistic cancer therapy. The nanomotor is constructed from bowl-shaped mesoporous polydopamine nanoparticles loaded with Fe(II) as a Fenton catalyst and BNN6 as a thermally responsive NO donor (denoted as PFB). To endow tumor specificity, the nanomotor is further camouflaged with MCF-7 cancer cell membrane (PFB@CM), enabling homologous recognition and enhanced intratumoral accumulation. Upon NIR irradiation, PFB@CM exhibits strong photothermal conversion efficiency that initiates 3 synergistic processes: (a) self-thermophoretic propulsion that promotes cellular internalization; (b) heat-triggered decomposition of BNN6 for precise NO release; and (c) heat-accelerated Fe(II) release from the polydopamine matrix. The liberated Fe(II) catalyzes endogenous H_2_O_2_ via a Fenton-like reaction to generate reactive oxygen species, which subsequently react with NO to yield highly cytotoxic reactive nitrogen species. This cascade amplifies oxidative and nitrosative stress within tumor cells, enabling photothermal, chemodynamic, and NO-mediated synergistic therapy. The design of PFB@CM integrates homologous targeting, autonomous motility, and NIR-responsive multimechanism activation, demonstrating a versatile strategy for precision nanomedicine and highlighting the potential of light-activated nanomotors for safe and effective multimodal cancer therapy.

## Introduction

Nanomotors have emerged as a versatile class of active nanotherapeutics capable of converting external energy inputs, such as magnetic fields, ultrasound, light, or chemical reactions, into directed motion [1–7]. Their autonomous propulsion enables enhanced cellular internalization and improved penetration through complex biological barriers, addressing the key limitations of passive nanocarriers [8–10]. In recent years, nanomotors driven by near-infrared (NIR) light have attracted particular attention due to their deep tissue penetration, precise spatiotemporal control, and compatibility with photothermal therapy (PTT). These nanomotors typically generate propulsion through thermophoresis, self-thermophoresis, or bubble-driven motion, thereby coupling active locomotion with localized photothermal heating [11–13]. This integrated functionality enables efficient traversal of mucus layers, extracellular matrices, and tumor interstitium, while simultaneously allowing multiple therapeutic modalities to be combined for synergistic anticancer outcomes [14–16]. For example, Wang et al. [12] developed a self-thermophoretic nanomotor that penetrated intestinal mucus and epithelial barriers, achieving a 3.5-fold enhancement in drug delivery efficiency. Xing et al. [17] demonstrated that NIR-powered nanomotors substantially improve cellular uptake and 3-dimensional tumor penetration, leading to robust tumor suppression by combining PTT with chemodynamic therapy (CDT). Despite these advances, the translating nanomotors into effective in vivo therapeutic systems remains challenging due to issues such as insufficient responsiveness, uncontrolled drug release, rapid clearance by the immune system, and limited specificity for tumor tissues [18,19]. Addressing these issues is essential for enabling nanomotors to function as precise, safe, and efficient carriers that actively deliver therapeutic payloads to diseased tissues [20,21].

Nitric oxide (NO) is a key endogenous signaling molecule that regulates diverse physiological and pathological processes [22–24]. At high concentrations (>1 μM), NO exhibits potent cytotoxic effects against cancer cells by inducing DNA strand breaks, inhibiting DNA synthesis and repair, promoting protein nitrosation, and activating apoptotic pathways [25–27]. In addition, NO can readily react with reactive oxygen species (ROS) to generate highly toxic reactive nitrogen species (RNS), such as peroxynitrite (ONOO^−^), which further amplifies oxidative and nitrosative damage in cancer cells [28,29]. Despite these therapeutic advantages, effective NO delivery remains challenging owing to its extremely short half-life (5 s), limited diffusion range (40 to 200 μm), and biphasic concentration-dependent effects in which submicromolar NO levels (10^−3^ to 10^−6^ mM) may inadvertently promote tumor growth rather than inhibit it [22]. These constraints highlight the necessity for strategies that enable selective tumor accumulation and precise spatiotemporal control of NO release to ensure safety and therapeutic efficacy [30,31]. To overcome these challenges, numerous NO delivery platforms have been designed, such as 2-dimensional nanomaterials [32], metal–organic frameworks [33], and polymeric micelles [34]. However, most existing systems rely primarily on passive accumulation through the enhanced permeability and retention effect, resulting in limited tumor penetration, inadequate selectivity, and suboptimal control of NO release. Consequently, their NO delivery efficiency and overall therapeutic precision remain unsatisfactory.

CDT has emerged as a complementary strategy that exploits endogenous H_2_O_2_ in the tumor microenvironment to generate cytotoxic ROS via Fenton-like reactions catalyzed by transition metal ions [35,36]. Among these catalysts, Fe(II) is particularly attractive due to its high Fenton reactivity [37,38]. However, the nonspecific distribution of Fe(II) in vivo may cause off-target effects, potentially causing systemic oxidative stress [39]. Therefore, the development of intelligent drug delivery platforms that enable tumor-specific accumulation and controlled release of catalytic species is crucial for enhancing therapeutic efficacy while minimizing systemic toxicity.

To address the challenges of poor targeting, rapid immune clearance, and limited efficacy associated with conventional nanotherapies, we developed a NIR-driven biomimetic nanomotor platform that integrates PTT, CDT, and NO therapy. This design aims to synergistically enhance tumor-specific targeting, deep tissue penetration, and multimodal therapeutic efficacy. Herein, we report the development of a biomimetic nanomotor, PFB@CM, constructed by loading bowl-shaped mesoporous polydopamine (PDA) nanoparticles with Fe(II) as a Fenton catalyst and BNN6 as a thermally labile NO donor, followed by cloaking with the MCF-7 cell membrane. Cell membrane camouflage reduces recognition and clearance by the immune system while enabling homologous targeting via native membrane proteins, thereby improving tumor homing and intratumoral retention [40]. Under NIR laser irradiation, PFB@CM exhibits a strong photothermal response that induces self-thermophoretic propulsion, substantially enhancing cellular uptake while simultaneously triggering the heat-induced decomposition of BNN6 for on-demand NO release. Within the tumor microenvironment, PFB@CM releases Fe(II) ions that catalyze endogenous H_2_O_2_ via the Fenton reaction to produce highly toxic hydroxyl radicals (·OH), thus initiating CDT. The photothermal effect further amplifies therapeutic efficiency by producing localized hyperthermia that (a) directly induces tumor cell apoptosis and (b) accelerates Fe(II) release, resulting in intensified ROS production. Notably, ROS generated in situ can further react with NO to form more potent RNS, establishing a cascade amplification mechanism that elevates oxidative and nitrosative stress. This multimodal therapeutic strategy, combining homologous targeting, active propulsion, photothermal heating, and synergistic ROS/RNS generation, demonstrated enhanced antitumor efficacy in MCF-7 tumor-bearing mouse models. The NIR-driven biomimetic nanomotor PFB@CM represents a versatile platform for targeted cancer therapy, offering a novel paradigm for multimodal synergistic treatment.

## Materials and Methods

### Materials

Dopamine hydrochloride, ammonia solution (NH_3_·H_2_O), 1,3,5-trimethylbenzene, 3,3',5,5'-tetramethylbenzidine (TMB) and hydrogen peroxide (H_2_O_2_) were obtained from Aladdin (China). Pluronic F-127 was obtained from Sigma-Aldrich. Ferrous sulfate heptahydrate (FeSO_4_·7H_2_O) was purchased from Macklin (China). 2,7-Dichlorodihydrofluorescein diacetate (DCFH-DA) was sourced from MedChem Express (USA). 3-Amino,4-aminomethyl-2',7'-difluorescein, diacetate (DAF-FM DA) was purchased from Biosharp (China), and ONOO^−^ probe was purchased from Bestbio (China). Dulbecco’s modified eagle medium (DMEM) was purchased from Gibco (USA).

### Synthesis of bowl-shaped PDA nanoparticles

Dopamine hydrochloride (1.5 g) and 1 g of Pluronic F-127 were mixed in 50 mL of deionized (DI) water and 50 mL of ethanol. Then, 3 mL of 1,3,5-trimethylbenzene was introduced while stirring, and the mixture was sonicated for 5 min to create an emulsion. Next, 3 mL of NH_3_·H_2_O (28 wt%) was added slowly while stirring at 800 rpm. After 2 h, the product was separated by centrifugation, washed with DI water and sonicated in acetone for 1 h to remove the F-127 template. To prepare PDA-Fe, 10 mg of bowl-shaped PDA nanoparticles were dispersed in 20 mL of DI water, followed by the addition of 200 mg of FeSO_4_·7H_2_O. The mixture was stirred for 4 h, centrifuged, and washed thoroughly with DI water to yield the PDA-Fe nanoparticles.

### Synthesis of PDA-Fe/BNN6 nanoparticles

In a 10-mL ethanol solution, 10 mg of PDA-Fe and 10 mg of BNN6 were dispersed and stirred at room temperature overnight. The mixture was centrifuged at 8,000 rpm, after which the resulting precipitate was washed to produce PDA-Fe/BNN6 (PFB) nanoparticles. The final product was dispersed in DI water, which had been purged with N_2_ for 30 min, and stored at 4 °C.

### Synthesis of PFB@CM nanomotors

The MCF-7 cell membranes were ultrasonically dispersed in an ice bath for 5 min, followed by the addition of 200 μL of PFB dispersion (1 mg/mL). The mixed solution was ultrasonicated in an ice bath for 10 min, and the excess cell membrane was removed by centrifugation at 8,000 rpm for 10 min to obtain cell membrane-coated PFB (PFB@CM).

### Chemodynamic properties of PFB

The samples’ Fenton-like activity was assessed using the conventional colorimetric method based on TMB oxidation. In a typical procedure, a reaction mixture containing the sample (200 ppm), H_2_O_2_ (1 mM), and TMB (20 mM) was prepared to a volume of 1 mL. Following a 30-min period, the oxidized TMB was characterized by imaging and ultraviolet–visible (UV–vis) absorption spectroscopy.

### Detection of extracellular NO generation

DAF-FM DA (2 μL, 5 mM) was hydrolyzed in 100 μL of NaOH (10 mM) for 30 min to generate DAF-FM, followed by reaction termination with 100 μL of phosphate-buffered saline (PBS). Subsequently, 100 μL of the resulting DAF-FM solution was mixed with 1 mL of the PFB-containing solution. The PFB + NIR group was irradiated with NIR (808 nm, 1.0 W/cm^2^) for 10 min. After incubating for 2 h at 37 °C, the fluorescence intensity was measured using fluorescence spectrometry. The PFB dispersions (50, 100, 150, and 200 ppm) were irradiated (808-nm NIR, 1.0 W/cm^2^) for 10 min and centrifuged to collect the supernatant. To detect NO, 100 μL of supernatant was combined with an equal amount of Griess reagent. The mixture was then kept in the dark for 20 min, following which the absorbance was measured at 540 nm by a microplate reader. NO concentrations were assessed using a standard curve.

### Determination of ONOO^–^ generation

ONOO^−^ probe (10 μL) was added to the solutions of control, PFB, PFB + H_2_O_2_, PFB + NIR, and PFB + H_2_O_2_ + NIR groups, respectively. Following a 2-h incubation at 37 °C, the fluorescence signal was measured by fluorescence spectroscopy.

### Detection of intracellular ROS

MCF-7 cells were placed into 96-well plates at a density of 1 × 10^4^ cells per well and cultured for 24 h. The cells underwent a series of treatments for 4 h: (a) PBS (control), (b) H_2_O_2_ (100 μM), (c) NIR (808 nm, 1.0 W/cm^2^, 10 min), (d) PFB@CM (100 ppm), (e) PFB@CM + H_2_O_2_, and (f) PFB@CM + H_2_O_2_ + NIR. Subsequently, the cells were incubated with DCFH-DA (10 μM in DMEM) for 20 min. Finally, the cells were rinsed with PBS. Fluorescence imaging was performed using an inverted fluorescence microscope.

### Detection of intracellular NO

MCF-7 cells were placed into 96-well plates at a density of 1 × 10^4^ cells per well and cultured for 24 h. Subsequently, the cells were treated with PBS, PFB@CM, and PFB@CM + NIR, respectively. After 4 h, the medium was discarded, and DAF-FM DA (5 μM) was introduced to stain the cells for 20 min, followed by a 10-min staining with Hoechst-33342. Fluorescence imaging was conducted by an inverted fluorescence microscope.

### Detection of intracellular ONOO^−^

MCF-7 cells were seeded in 96-well plates at a density of 1 × 10^4^ cells per well and cultured for 24 h. The cells were then treated for 4 h under the following conditions: (a) PBS (control); (b) PFB@CM; (c) PFB@CM + NIR; (d) PFB@CM + H_2_O_2_; and (e) PFB@CM + H_2_O_2_ + NIR. After treatment, the cells were incubated with a freshly prepared ONOO^−^ probe working solution in DMEM for 20 min. Finally, the cells were washed with PBS, and fluorescence imaging was performed using an inverted fluorescence microscope.

### Assessment of cell therapy efficacy

MCF-7 cells were placed into 96-well plates at a density of 1 × 10^4^ cells per well and cultured for 24 h. Following the removal of the initial medium, the cells underwent various treatments: PBS, NIR (1.0 W/cm^2^, 10 min), PFB@CM (100 ppm), PFB@CM + H_2_O_2_, PFB@CM + NIR, and PFB@CM + H_2_O_2_ + NIR. The cells were then incubated for another 24 h, and their viability was evaluated using the Cell Counting Kit-8 (CCK-8) assay. To visualize cell viability, the cells were stained with calcein-AM and propidium iodide (PI) for 20 min. After that, they were washed with PBS and observed using an inverted fluorescence microscope.

### Tumor models and in vivo tumor therapy

Female Balb/c nude mice (5 to 6 weeks) were obtained from Beijing Weitong Lihua Laboratory Animal Technology Co., Ltd. (China). The Animal Care and Use Committee of the Southern University of Science and Technology approved the animal experiments (Institutional Animal Care and Use Committee No. SUSTech-JY202502053). To establish a subcutaneous tumor model in mice, MCF-7 cells (4 × 10^6^ cells) were injected into the right leg of each mouse. The MCF-7 tumor-bearing mice were allocated into 6 groups through randomization, with each group comprising 5 mice: (a) Control; (b) PBS + NIR; (c) PFB@CM; (d) PDA@CM + NIR; (e) PDA-Fe@CM + NIR; and (f) PFB@CM + NIR. When the tumor volume was about 100 mm^3^, the mice were given an injection of PBS (100 μL) or respective drug formulations (100 μL, 2 mg/mL) through a vein. After 6 and 24 h postinjection, the mice were anesthetized and subjected to NIR laser irradiation (808 nm, 1.0 W/cm^2^) on the tumor region for 10 min. Body weight was recorded every 2 d to assess systemic toxicity. Tumor dimensions were taken using a caliper, and the volume of the tumor was determined with the formula: *V*_tumor_ = (tumor length) × (tumor width)^2^/2. After 14 d of treatment, the mice were euthanized, and their primary organs were collected for tissue analysis and staining.

### Statistical analysis

Quantitative data are presented as the means ± SD from at least 3 independent experiments. Statistical significance was analyzed using a 2-tailed Student *t* test. Differences with *P* < 0.05 were considered statistically significant, and significance levels are denoted as *P < 0.05, **P < 0.01, and ***P < 0.001.

## Results

### Synthesis and characterization of PFB@CM nanomotors

**Fig. 1.**
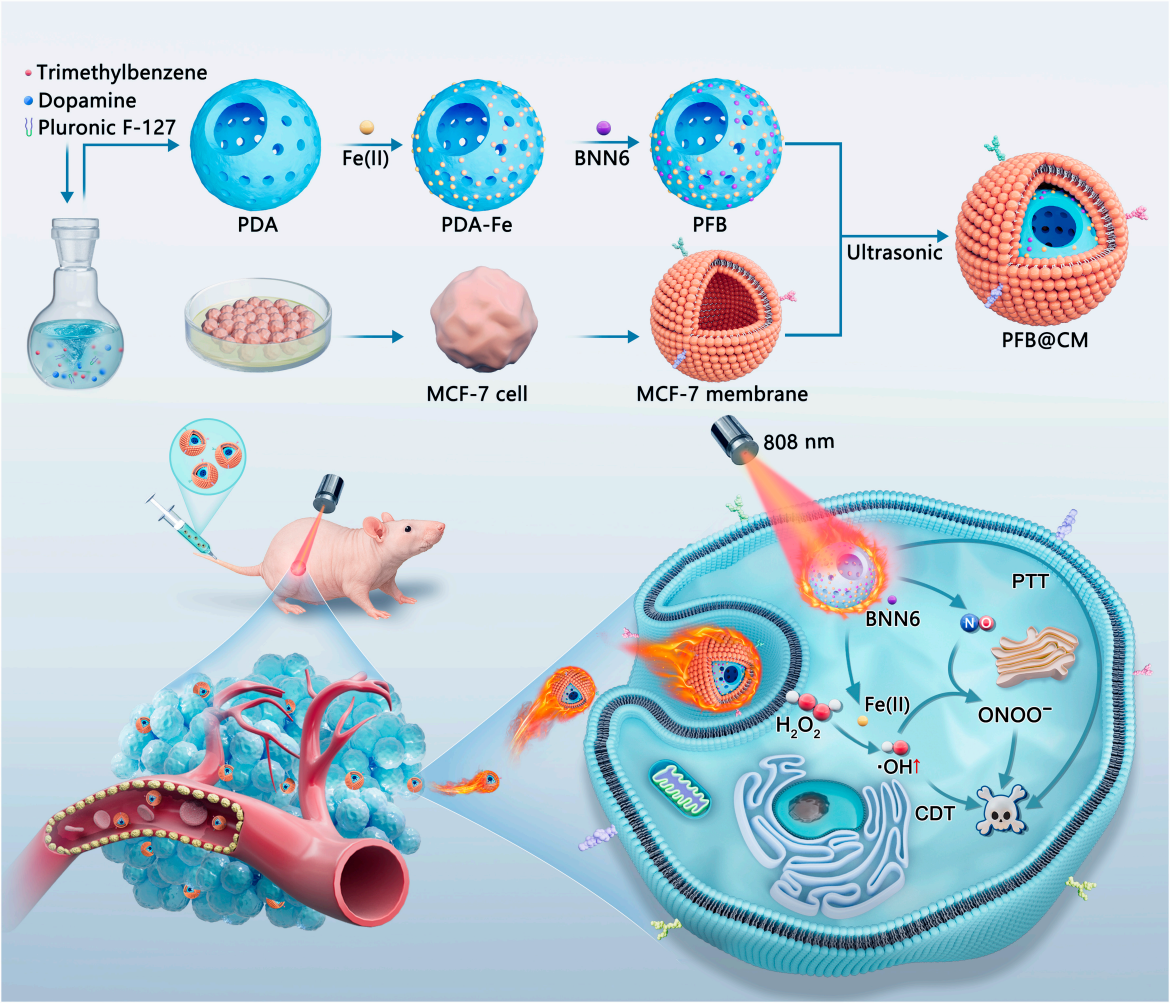
Schematic illustration of the fabrication of PFB@CM nanomotors and their near-infrared (NIR)-activated multimodal antitumor mechanisms involving photothermal, chemodynamic, and nitric oxide (NO)-mediated synergistic therapy. Polydopamine-Fe/BNN6 (PFB) nanoparticles were synthesized and camouflaged with cancer cell membranes to form PFB@CM nanomotors, thereby improving biocompatibility and tumor targeting. Under NIR irradiation, the PFB@CM nanomotors generated a strong photothermal response that drove 3 synergistic processes: self-thermophoretic propulsion to enhance cellular uptake, NO release via heat-triggered BNN6 decomposition, and accelerated Fe(II) liberation from the polydopamine (PDA) matrix. The released Fe(II) converts endogenous H_2_O_2_ into ·OH via a Fenton-like reaction, and ·OH further reacts with NO to yield cytotoxic ONOO^−^.

**Fig. 2.**
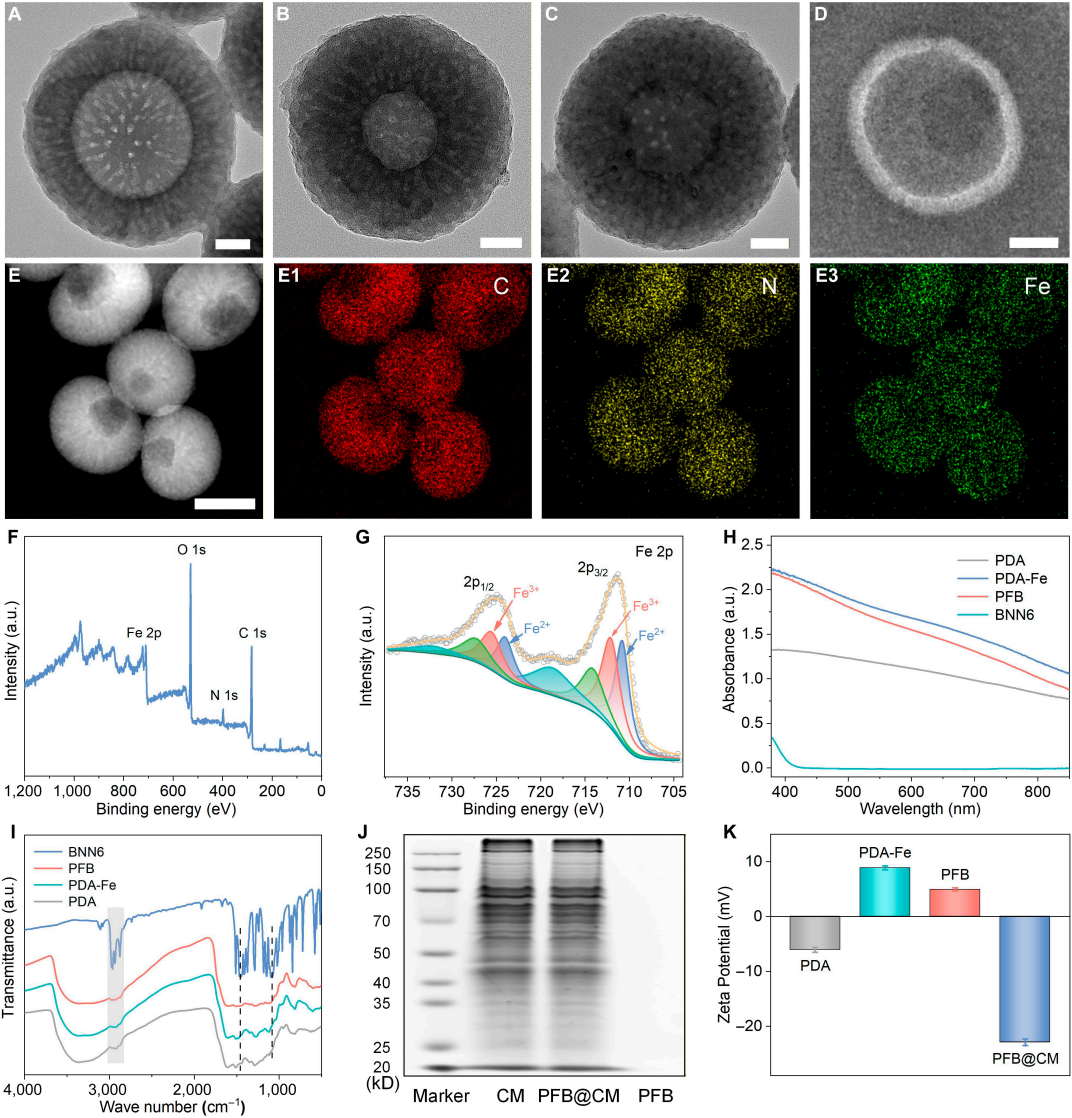
Synthesis and characterization of polydopamine-Fe/BNN6 (PFB) and PFB@CM nanomotors. Transmission electron microscopy (TEM) images of (A) polydopamine (PDA), (B) PDA-Fe, (C) PFB, and (D) negatively stained PFB@CM nanomotors. Scale bar: 50 nm. (E) STEM-high-angle annular dark-field (HAADF) image and corresponding elemental mappings of PFB (E1: C, E2: N, E3: Fe). (F) X-ray photoelectron spectroscopy (XPS) spectrum and (G) high-resolution Fe spectrum of PFB. (H) Ultraviolet–visible (UV–vis) absorption spectra of PDA, PDA-Fe, PFB, and BNN6. (I) Fourier-transform infrared (FTIR) spectra of BNN6, PDA, PDA-Fe, and PFB. (J) Sodium dodecyl sulfate-polyacrylamide gel electrophoresis (SDS-PAGE) analysis confirming the preservation of MCF-7 cell membrane proteins. (K) Zeta potentials of PDA, PDA-Fe, PFB, and PFB@CM. a.u., arbitrary units.

The fabrication of the biomimetic nanomotor PFB@CM is illustrated in Fig. 1. First, bowl-shaped PDA nanoparticles were synthesized according to a previously reported protocol [41], serving as a biocompatible carrier with a high loading capacity. Scanning electron microscopy and transmission electron microscopy (TEM) images of the synthesized PDA nanoparticles revealed uniform bowl-shaped structures with an average diameter of approximately 250 nm and distinct radially aligned mesoporous channels, providing ample space for loading therapeutic agents (Fig. 2A and Fig. S1). Owing to the abundant catechol groups on PDA, the PDA nanoparticles were first coordinated with Fe(II) ions to form PDA-Fe, followed by the incorporation of the thermally responsive NO donor BNN6 to yield PFB. The TEM images of PDA-Fe (Fig. 2B) and PFB (Fig. 2C) displayed increased electron-dense material within the mesopores and on the particle surface, confirming the successful sequential loading of Fe(II) and BNN6. To enable tumor-specific targeting and immune evasion, PFB nanoparticles were cloaked with MCF-7 cancer cell membrane fragments. Negative-staining TEM of PFB@CM (Fig. 2D) revealed a continuous, uniform shell surrounding the particle, confirming successful membrane encapsulation. High-angle annular dark-field TEM imaging (Fig. 2E) and the corresponding elemental mapping (Fig. 2e1 to e3) of the PFB nanoparticles demonstrated uniform distributions of carbon, nitrogen, and iron, validating the presence of PDA, BNN6, and Fe(II) ions throughout the nanomotor. X-ray photoelectron spectroscopy (XPS) analysis further confirmed the composition and chemical state of Fe. The survey spectrum (Fig. 2F) displays characteristic peaks for C 1s, N 1s, O 1s, and Fe 2p. The high-resolution Fe 2p XPS spectrum (Fig. 2G) revealed the presence of mixed-valence iron species on the surface of the PFB material. The spectrum exhibits 2 sets of peaks, assigned to Fe^2+^ (710.9 and 724.0 eV) and Fe^3+^ (712.2 and 725.8 eV), respectively. The coexistence of these oxidation states suggests partial oxidation of surface Fe^2+^ species. This is attributed to the strong reducibility of Fe^2+^, which leads to the surface oxidation of Fe^2+^ upon air exposure during sample handling, forming a thin surface layer of Fe^3+^ oxides/hydroxides. Consequently, the XPS signal originates predominantly from this oxidized surface region, consistent with the observed mixed Fe^2+^ and Fe^3+^ chemical state. The successful incorporation of BNN6 was further confirmed by UV–vis and Fourier-transform infrared spectroscopy. As shown in Fig. 2H, a characteristic absorption band of BNN6 was observed near 250 nm in the UV–vis spectrum, confirming successful incorporation, which was further supported by Fourier-transform infrared analysis (Fig. 2I) showing characteristic BNN6 vibrations retained in the PFB sample [32]. The successful camouflage of PFB nanoparticles with MCF-7 cell membranes was further assessed using sodium dodecyl sulfate-polyacrylamide gel electrophoresis. The results (Fig. 2J) indicate that the PFB@CM nanomotors retained a membrane protein profile highly consistent with that of the isolated native cell membranes, whereas no protein bands were detected for uncoated PFB nanoparticles. This confirms the effective translocation of cell membrane proteins to the nanomotor surface. Furthermore, the successful stepwise construction of the nanomotor was evidenced by a progressive shift in the zeta potential (Fig. 2K). The potential changed from −6.1 mV for bare PDA nanoparticles to +8.8 mV after Fe(II) loading, then to +4.9 mV after BNN6 incorporation, and finally to −22.9 mV after the membrane coating. This systematic shift reflects the successful assembly of the PFB@CM nanomotors and the presence of a negatively charged biomimetic membrane surface.

### Directed motion of PFB@CM nanomotors under NIR irradiation

**Fig. 3.**
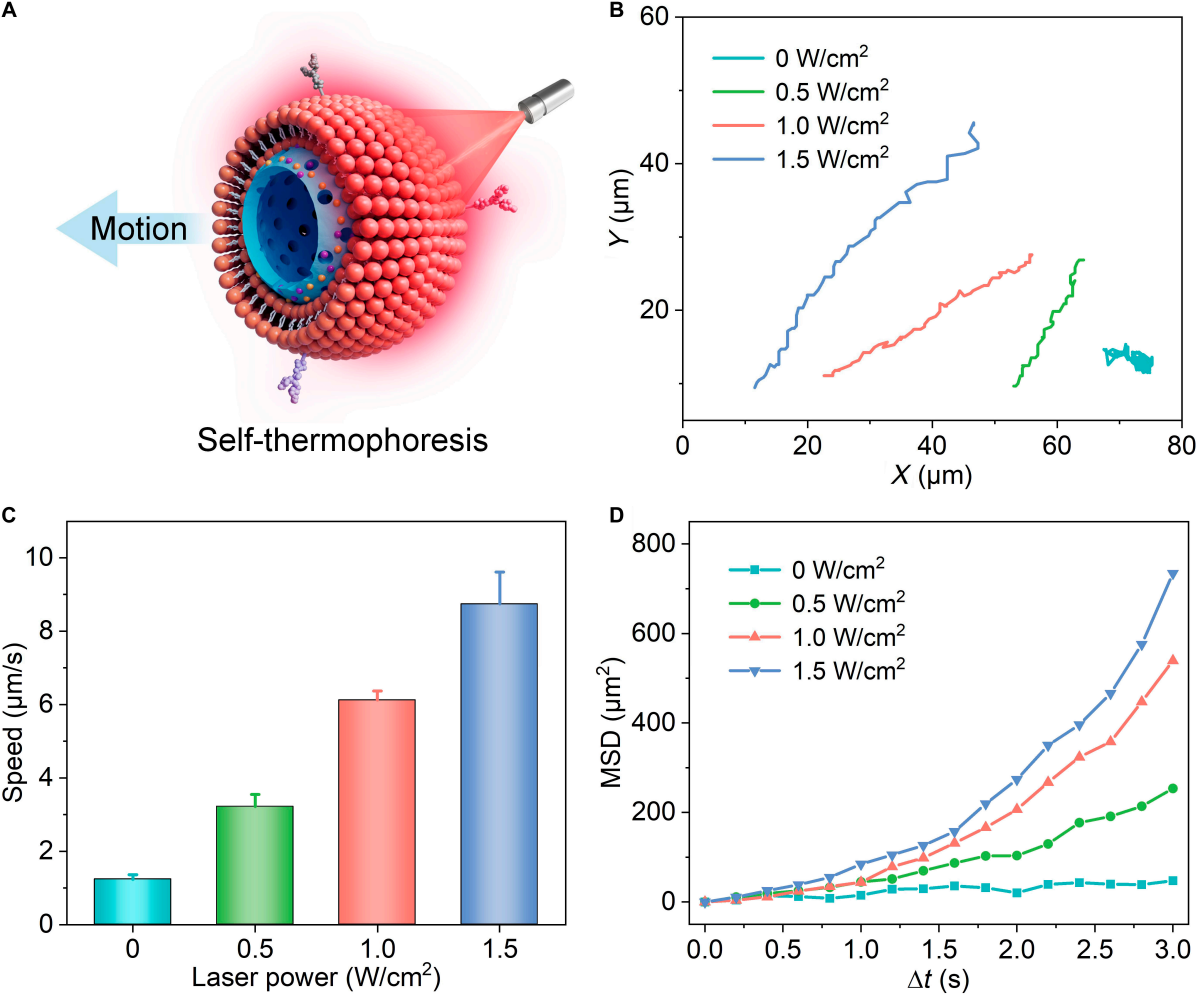
Near-infrared (NIR)-driven propulsion behavior of PFB@CM nanomotors. (A) Schematic illustration of the self-thermophoresis of the PFB@CM nanomotor under NIR irradiation. (B) Representative trajectories, (C) average propulsion speeds, and (D) mean-square displacement profiles of the PFB@CM nanomotors under NIR irradiation.

Self-thermophoresis is a key propulsion mechanism in which nanomotors generate a localized temperature field at their surfaces, thereby inducing motion in the surrounding fluid. This mechanism enables precise motion control at the nanomotors and holds considerable promise for applications such as targeted delivery. The photothermally driven self-thermophoretic effect relies on an asymmetric material/structural design to establish a pronounced temperature gradient. Under NIR laser irradiation, the Janus PFB@CM nanomotor exhibits a strong photothermal response, creating a distinct temperature gradient between its irradiated and nonirradiated sides (Fig. 3A). This thermal gradient drives directional movement from the hotter to the cooler region via self-thermophoresis [42]. The representative motion trajectories of the PFB@CM nanomotor at different NIR power densities are shown in Fig. 3B. In the absence of NIR light, the nanomotors exhibited random Brownian motion, whereas directed movement was observed upon irradiation. The velocity of the PFB@CM nanomotors increased with increasing NIR power density, accompanied by enhanced directionality, which collectively confirmed their NIR-driven propulsion capability. Specifically, as the power density increased from 0.5 to 1.5 W/cm^2^, the average velocity of the PFB@CM nanomotors increased from 3.2 to 8.7 μm/s (Fig. 3C). This acceleration effect can be attributed to the enhanced temperature gradient across the nanoparticle surface at higher power densities, which improves the self-thermophoretic propulsion efficiency of the nanomotors. Consistently, the mean square displacement exhibited a parabolic increase over time (Fig. 3D), indicating the presence of effective directional propulsion.

### Photothermal conversion and extracellular catalytic performance

**Fig. 4.**
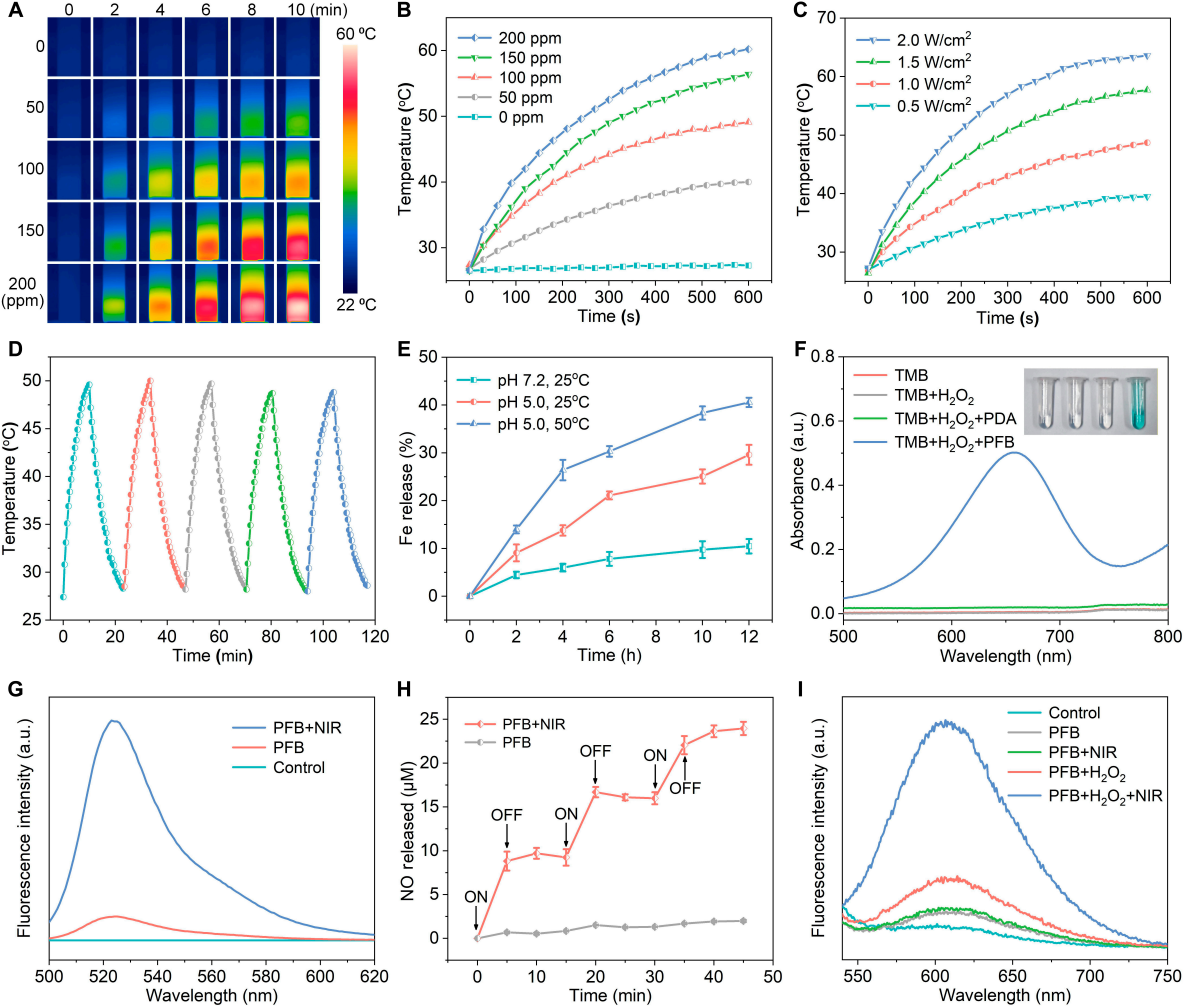
Photothermal behavior and extracellular catalytic performance of polydopamine-Fe/BNN6 (PFB). (A) Thermal images of PFB at varying concentrations in quartz cuvettes under 808-nm laser irradiation (1.0 W cm^−2^). (B) Corresponding temperature–time profiles. (C) Photothermal heating curves of PFB under 808-nm laser irradiation at different power densities. (D) Photothermal stability of PFB over 5 laser on/off cycles. (E) Fe ion release from PFB at various pH levels and temperatures. (F) Ultraviolet–visible (UV–vis) absorption spectra of oxidized 3,3',5,5'-tetramethylbenzidine (oxTMB) for different experimental groups. (G) Fluorescence spectra of 3-amino,4-aminomethyl-2',7'-difluorescein diacetate (DAF-FM DA) after near-infrared (NIR) laser irradiation of PFB. (H) Nitric oxide (NO) release rate of PFB nanoparticles with or without intermittent NIR irradiation. (I) Production of ONOO^−^ via cascade reactions of NO and reactive oxygen species (ROS). a.u., arbitrary units.

Given the pivotal role of PFB@CM nanomotors in PTT and NO generation, we first evaluated their photothermal conversion properties. UV–vis spectral analysis (Fig. 2H) showed negligible absorption of BNN6 at 808 nm, whereas PDA-Fe exhibited strong absorption after Fe(II) coordination with the PDA matrix. Fig. 4A shows infrared thermal images of PFB suspensions at various concentrations (0, 50, 100, 150, and 200 ppm) under 808-nm laser irradiation at a power density of 1.0 W/cm^2^, which demonstrated a clear concentration-dependent photothermal response. Correspondingly, the temperature elevation increased from 1.2 °C for pure water to 33.6 °C for 200-ppm PFB over 10 min of irradiation (Fig. 4B). Notably, the temperature of the 100-ppm PFB suspension increased by 21.7 °C within 10 min, which was sufficient to inhibit cancer cell growth and trigger NO release. The photothermal effect was also power density-dependent (0.5 to 2.0 W/cm^2^) and remained stable over 5 laser on/off cycles (Fig. 4C and D), highlighting the robust photothermal performance of the PFB@CM nanomotors.

The controlled release of Fe(II) from the nanomotor is crucial for activating Fenton-like reactions. To simulate the tumor microenvironment (pH 5.0) and physiological conditions (pH 7.4), we monitored Fe(II) release in buffers with different pH values. As shown in Fig. 4E, an acidic pH markedly​ accelerated Fe(II) release owing to the protonation-induced weakening of the Fe-PDA coordination bonds. Furthermore, elevated temperature (50 °C) mimicking photothermal conditions further enhanced Fe(II) dissociation, consistent with thermally promoted bond cleavage and increased diffusion. In the tumor microenvironment, the released Fe(II) ions catalyze Fenton-like reactions with endogenous H_2_O_2_ to yield cytotoxic ·OH radicals [39]. The catalytic performance of the PFB@CM nanomotors was assessed using TMB as an ·OH probe. Incubation of PFB nanoparticles with TMB and H_2_O_2_ produced a distinct absorption peak at 650 nm, accompanied by a visible color change from colorless to blue, confirming their strong Fenton-like activity (Fig. 4F). The ·OH generation efficiency was further verified via the methylene blue (MB) decolorization assay. As demonstrated in Fig. S2, in contrast to treatments with PFB or H_2_O_2_ alone, the characteristic absorption peak of MB completely disappeared in the PFB + H_2_O_2_ group, confirming the efficient degradation of MB by ·OH. A time-dependent decrease in MB absorbance (Fig. S3) further indicates that PFB continuously catalyzes H_2_O_2_ decomposition to produce ·OH.

To evaluate photothermal-triggered NO generation, we employed the fluorescence probe DAF-FM DA for NO detection. Fluorescence detection (Fig. 4G) showed no signal in the control group and only weak fluorescence in the PFB group, attributable to the slow spontaneous decomposition of BNN6. However, upon NIR irradiation, the PFB group displayed a pronounced increase in fluorescence intensity, confirming that PDA-induced photothermal heating effectively accelerated NO release from BNN6. Quantitative analysis using the Griess assay (Fig. 4H) further demonstrated the regulatory effect of the NIR laser on NO release. Upon NIR irradiation, PFB-released NO was detected at concentrations of up to 8.8 μM. The NO concentration showed no apparent change after the laser was turned off, whereas a marked increase in release was observed upon reapplying the NIR laser. In contrast, the PFB control group without NIR irradiation produced only a negligible amount of NO, indicating that NO release from PFB is highly photothermal responsive. Moreover, under fixed irradiation conditions, NO generation increased in a concentration-dependent manner with increasing PFB concentration (Fig. S4). Together, these results demonstrate that the photothermal conversion capability of PDA can effectively trigger the decomposition of loaded BNN6, thereby enabling on-demand-NO release. Given that NO can react with ROS to produce ONOO^−^ with enhanced cytotoxicity, ONOO^−^ levels were evaluated using a peroxynitrite-responsive fluorescent probe. As shown in Fig. 4I, the PFB + H_2_O_2_ group displayed a moderate increase in fluorescence, which was attributed to the partial cross-reactivity of the probe with ROS. In contrast, the PFB + H_2_O_2_ + NIR group showed stronger fluorescence upon NIR irradiation, confirming efficient ONOO^−^ generation via the cascade reaction between photothermally triggered NO and ROS produced through the Fenton-like process.

### Intracellular catalytic performance

**Fig. 5.**
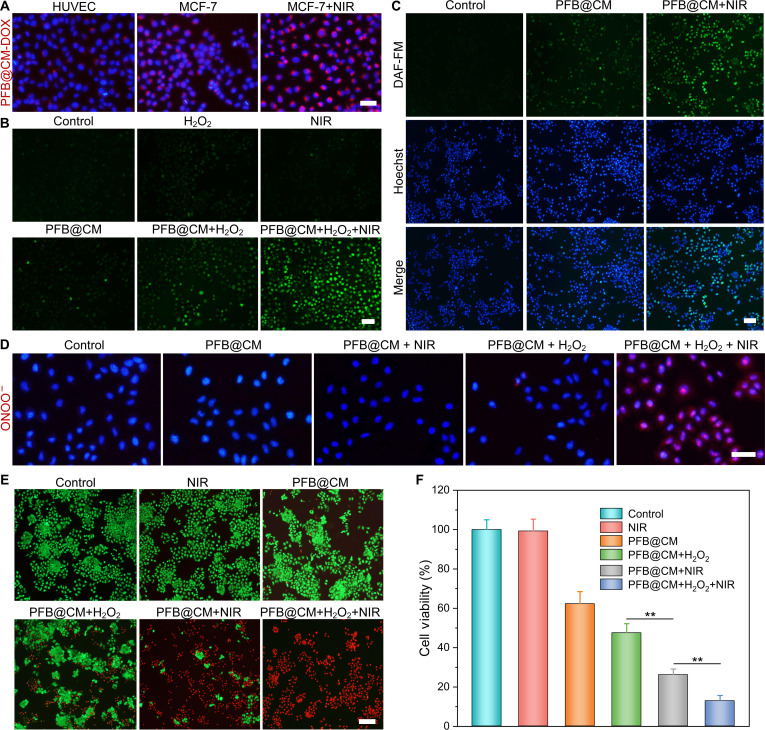
In vitro catalytic activities and cancer cell inhibition effects of PFB@CM nanomotors. (A) Fluorescence images of human umbilical vein endothelial cell (HUVEC) and MCF-7 cells following a 4-h incubation with doxorubicin (DOX)-labeled PFB@CM. Scale bar: 50 μm. (B) Detection of intracellular reactive oxygen species (ROS) production in MCF-7 cells using the 2,7-dichlorodihydrofluorescein diacetate (DCFH-DA) probe. Scale bar: 100 μm. (C) Detection of intracellular nitric oxide (NO) production by 3-amino,4-aminomethyl-2',7'-difluorescein diacetate (DAF-FM DA) staining. Scale bar: 100 μm. (D) Detection of ONOO^−^ levels in MCF-7 cells staining. Scale bar: 50 μm. (E) Live/dead staining (calcein-AM/propidium iodide [PI]) of MCF-7 cells treated under the indicated conditions (Control, near-infrared [NIR], PFB@CM, PFB@CM + H_2_O_2_, PFB@CM + NIR, and PFB@CM + H_2_O_2_ + NIR). Scale bar: 200 μm. (F) Quantified viabilities of MCF-7 cells under corresponding treatments. Statistical significance was calculated by Student 2-tailed *t* test (**P < 0.01).

The PFB@CM nanomotor exhibited efficient NIR-driven propulsion and photothermal-assisted generation of ROS, NO, and RNS in extracellular experiments. Following camouflage with MCF-7 cancer cell membranes, the PFB@CM nanomotors displayed improved biocompatibility and tumor-targeting capability. To assess cellular uptake efficiency, doxorubicin (DOX)-loaded PFB@CM nanomotors were incubated with MCF-7 and human umbilical vein endothelial cells (HUVEC) cells. Fluorescence microscopy (Fig. 5A) revealed significantly stronger DOX signals in MCF-7 cells compared to HUVEC cells, confirming the homologous targeting capability conferred by the MCF-7 cell membrane coating. Notably, NIR irradiation (0.5 W/cm^2^, 30 min) further enhanced nanomotor uptake in MCF-7 cells, attributable to photothermally induced self-thermophoresis.

Intracellular ROS generation was monitored utilizing the DCFH-DA fluorescent probe. As shown in Fig. 5B, MCF-7 cells incubated with PFB@CM and H_2_O_2_ for 4 h exhibited distinct​ green fluorescence, confirming the production of intracellular ROS. This signal was further intensified under NIR irradiation (808 nm, 1.0 W/cm^2^, 10 min), reflecting thermally accelerated Fe(II) release that enhances the Fenton-like reaction and subsequent ·OH generation. The elevated ROS levels can induce irreversible cellular damage through lipid peroxidation, contributing to the potent antitumor efficacy of PFB@CM nanomotors. Intracellular NO release was examined using the NO-sensitive probe DAF-FM DA, with nuclei stained with Hoechst. PFB@CM-treated cells showed weak green fluorescence, whereas NIR irradiation triggered a dramatic increase in fluorescence (Fig. 5C), demonstrating that photothermal heating effectively activates BNN6 decomposition for controlled intracellular NO release. To evaluate intracellular ONOO^−^ formation, a peroxynitrite-specific fluorescent probe was employed. As shown in Fig. 5D, PFB@CM-treated MCF-7 cells, with or without NIR irradiation, exhibited minimal fluorescence, indicating negligible ONOO^−^ formation in the absence of sufficient ROS. The addition of H_2_O_2_ to PFB@CM-treated cells induced negligible red fluorescence, consistent with intracellular ROS production. In contrast, an intense red fluorescence was detected in cells following cotreatment with PFB@CM, H_2_O_2_, and NIR irradiation (808 nm, 1.0 W/cm^2^, 10 min), clearly demonstrating efficient ONOO^−^ generation via the reaction of photothermally released NO with Fenton-derived ·OH.

Leveraging the multimodal functionalities of PFB@CM nanomotors, we evaluated their in vitro anticancer performance against MCF-7 cancer cells. MCF-7 and HUVEC cells were treated with varying concentrations of PFB@CM, and their viability was determined using the CCK-8 assay (Fig. S5). The results revealed a concentration-dependent cytotoxicity against MCF-7 cells, accompanied by lower toxicity toward HUVECs. This selective cytotoxicity arises from the homologous targeting capability conferred by the MCF-7 cell membrane coating, which promotes the preferential uptake of PFB@CM by the source cancer cells. To further assess the therapeutic performance, cell apoptosis was examined using calcein-AM/PI live/dead staining and CCK-8 analysis (Fig. 5E and F). NIR laser irradiation alone (808 nm, 1.0 W/cm^2^, 10 min) showed negligible inhibitory effects on MCF-7 cells, whereas PFB@CM (100 ppm) treatment achieved 36.8% inhibition of cancer cell viability. In comparison, combining PFB@CM with either H_2_O_2_ or NIR irradiation resulted in increased PI fluorescence and a significant reduction in cell viability, underscoring the contributions of ROS-mediated CDT and photothermal effects. Notably, the PFB@CM + H_2_O_2_ + NIR group exhibited the strongest anticancer efficacy, characterized by minimal green calcein-AM staining and a remarkable inhibition rate of 87.2%. This potent cytotoxic response provides clear evidence of the synergistic therapeutic outcome, confirming the synergistic interplay of PTT and ROS/RNS-mediated oxidative stress, resulting in significantly enhanced anticancer efficacy compared to monotherapy.

### In vivo multimodal antitumor performance

**Fig. 6.**
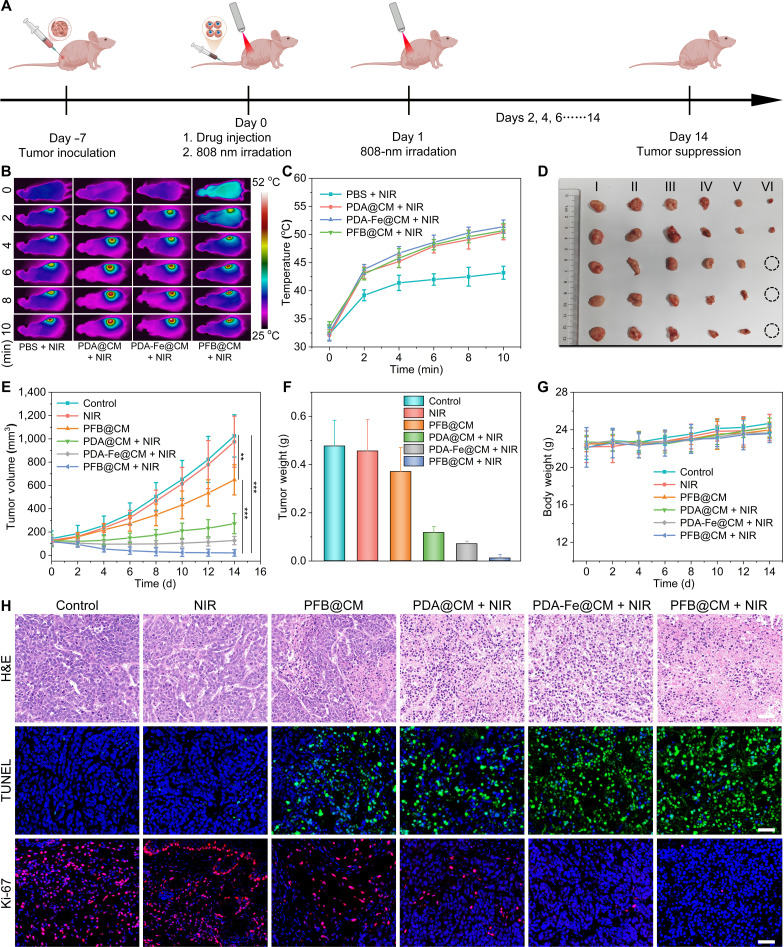
In vivo antitumor evaluation of PFB@CM nanomotors. (A) Schematic illustration of the treatment protocol using MCF-7 tumor-bearing nude mice. (B) Thermal images and (C) recorded temperature profiles of tumor sites under 808-nm irradiation at 6 h postinjection. (D) Photographs of excised tumors collected from each treatment group at the study end point (*n* = 5). (E) Tumor growth curves after different treatments (*n* = 5). Statistical significance was calculated by Student 2-tailed *t* test (**P < 0.01, ***P < 0.001). (F) Corresponding tumor weights following different treatments (*n* = 5). (G) Body weight changes of mice monitored throughout the therapeutic period (*n* = 5). (H) Representative hematoxylin and eosin (H&E), terminal deoxynucleotidyl transferase biotin-dUTP nick end labeling (TUNEL), and Ki-67 staining of tumor sections at 24 h posttreatment, showing treatment-induced apoptosis and proliferation inhibition. Scale bar: 50 μm.

Encouraged by the potent multimodal cytotoxicity observed in vitro, the therapeutic performance of the PFB@CM nanomotors was further assessed in MCF-7 tumor-bearing mice. As illustrated in Fig. 6A, the mice were intravenously administered different formulations and subjected to external NIR laser irradiation (808 nm, 1.0 W/cm^2^, 10 min) at 6 and 24 h postinjection. Thermal imaging monitoring was employed to capture the temperature variation profile at the tumor site. As shown in Fig. 6B and C, the PBS + NIR group exhibited only a minimal temperature rise to 43.2 °C, whereas hyperthermia was observed in the PDA@CM + NIR (50.4 °C), PDA-Fe@CM + NIR (51.4 °C), and PFB@CM + NIR (50.7 °C) groups. These results demonstrate that PFB@CM nanomotors effectively accumulate in tumor tissues and exhibit exceptional photothermal properties. This localized hyperthermia not only provides the energy required for self-thermophoretic propulsion but also enables controlled NO release from BNN6, facilitating multimodal therapy.

To evaluate treatment efficacy, tumor volumes and mouse body weights were monitored every other day throughout the 14-d treatment period. In Fig. 6D to F, the PBS + NIR group showed negligible tumor suppression compared to the control group. PFB@CM monotherapy induced substantial tumor suppression, attributable to Fe(II)-mediated Fenton catalysis and ROS generation. NIR irradiation significantly enhanced tumor inhibition in all the treatment groups. The PDA-Fe@CM + NIR group displayed improved therapeutic performance, consistent with thermally accelerated CDT via enhanced Fenton kinetics. Notably, the PFB@CM + NIR group achieved superior therapeutic efficacy, with the final tumor volume reduced to 20.6 mm^3^ and tumor weight to 11.4 mg, representing 98.0% and 97.6% reductions compared to the control group, respectively. Tumor photographs and gravimetric analysis of excised tumors on day 14 provided further corroboration of these findings. Importantly, no significant body weight loss was observed throughout the treatment period (Fig. 6G), indicating minimal systemic toxicity. To elucidate the synergistic anticancer mechanisms of PTT and RNS/ROS therapy, tumor tissues were harvested 24 h posttreatment for histopathological analysis, including hematoxylin and eosin (H&E) staining, terminal deoxynucleotidyl transferase biotin-dUTP nick end labeling assay, and Ki-67 immunofluorescence (Fig. 6H). H&E staining revealed distinct morphological changes among the treatment groups. Compared with the control group, the NIR-irradiated treatment groups (PDA@CM + NIR, PDA-Fe@CM + NIR, and PFB@CM + NIR) exhibited cellular atrophy and tissue architectural disruption. Notably, the PFB@CM + NIR group demonstrated the most severe tumor damage, confirming the superior efficacy of combined PTT and RNS/ROS therapy. Terminal deoxynucleotidyl transferase biotin-dUTP nick end labeling staining, employed for the detection of apoptotic cells, demonstrated significantly green fluorescence signals in the PFB@CM-treated groups compared to the control and NIR groups, indicating effective cell apoptosis through Fenton reaction-mediated CDT. Further intensification of fluorescence was observed in both the PDA-Fe@CM + NIR and PFB@CM + NIR groups, demonstrating that the synergistic interaction between PTT and CDT substantially promoted tumor cell apoptosis. Complementary Ki-67 staining, used to assess cellular proliferation activity, showed abundant red fluorescence in the control and NIR groups, whereas the combined treatment groups, particularly the PFB@CM + NIR group, exhibited minimal proliferative signals, confirming the effective suppression of tumor cell proliferation.

These results collectively confirm that PFB@CM nanomotors function as a trifunctional therapeutic platform: serving as an efficient photothermal agent for PTT, a Fenton reaction catalyst for CDT, and a NIR-triggered NO donor enabling in situ generation of cytotoxic RNS through cascade reactions. To evaluate the biosafety of the PFB@CM nanomotors, we collected major organs (including the liver, spleen, kidneys, heart, and lungs) at the study end point for subsequent histopathological examination using H&E staining. The results (Fig. S6) demonstrated no detectable structural abnormalities or inflammatory lesions in any of the treatment groups, confirming the favorable in vivo biocompatibility of PFB@CM. Collectively, these results demonstrate that PFB@CM-mediated synergistic therapy, integrating PTT and ROS/RNS generation, achieves potent antitumor efficacy while maintaining an excellent safety profile in tumor-bearing mice.

## Discussion

Compared to single-modal cancer therapies, multimodal synergistic treatments offer enhanced efficacy and reduced drug resistance by combining multiple therapeutic mechanisms. This study presents a NIR-driven PFB@CM nanomotor that integrates PTT, CDT, and NO therapy on a single platform. The experimental results demonstrated that PFB@CM exhibited efficient photothermal conversion performance. Under 1.5 W/cm^2^ NIR light irradiation, it achieved a motion speed of 8.7 μm/s (Fig. 3C), exhibiting self-thermophoresis-driven motion that facilitates the penetration of physiological barriers and enhances cellular uptake. After 10 min of NIR laser exposure, a PFB dispersion (100 ppm) reached 49 °C (Fig. 4B), enabling direct tumor cell apoptosis via thermal ablation [43]. Photothermal triggering decomposed the thermoresponsive BNN6 donor to release NO, attaining a concentration of 8.8 μM within 5 min (Fig. 4H) and exhibiting potent cytotoxicity. Additionally, the acidic tumor microenvironment and photothermal effects accelerate Fe(II) release, catalyzing endogenous H_2_O_2_ to generate cytotoxic ROS for CDT. Subsequent reactions between NO and ROS produced more cytotoxic RNS, synergistically amplifying oxidative and nitrosative stress in tumor cells. This active targeting and multimodal synergistic mechanism exhibited potent antitumor efficacy. In vivo experiments demonstrated that PFB@CM combined with NIR laser irradiation reduced tumor volume and weight by 98.0% and 97.6% (Fig. 6F), respectively, while exhibiting excellent biocompatibility and no significant systemic toxicity.

PFB@CM offers several advantages over existing nanomotors. Most reported NIR-propelled nanomotors primarily rely on the enhanced permeability and retention effect for passive tumor accumulation, followed by photothermal-driven barrier penetration, which limits their delivery efficiency [11,17]. In contrast, PFB@CM employs a membrane camouflage strategy that confers both homologous targeting and immune evasion capabilities. The retained adhesion proteins and antigens from MCF-7 cell membrane enable specific recognition and binding to homologous cancer cells, achieving active targeting superior to passive targeting. After PFB@CM autonomously aggregates in the tumor region, its self-thermophoresis propulsion further facilitates penetration through biological barriers, improving tissue penetration efficiency.

Although cell membrane-modified nanomotors often enhance barrier penetration and drug delivery efficiency, they generally lack synergistic enhancement for multimodal therapies [12]. By integrating multiple therapeutic, PFB@CM achieves synergistic enhancement effects, effectively addressing common issues such as drug resistance and insufficient efficacy in monotherapies. For NO gas therapy, precise targeted delivery and controlled release are critical. Traditional NO donors often release spontaneously and lack spatiotemporal control. PFB@CM achieves specific tumor enrichment through homologous targeting, and the loaded BNN6 release NO precisely upon NIR laser activation. The release is synchronized with photothermal heating and ROS generation, creating optimal conditions for forming highly cytotoxic RNS.

The antitumor efficacy of PFB@CM stems from the synergistic between its autonomous targeting and multimodal therapy. Tumor-specific targeting ensures accumulation at tumor sites, while self-thermophoresis enhances penetration through biological barriers, overcoming the limitations of passive delivery. Simultaneously, photothermal effects increase cell membrane permeability, promoting cellular internalization. The synergistic multimodal action of PTT, NO, and ROS/RNS makes it difficult for tumor to develop resistance through a single mechanism. To ensure biosafety, the PDA carrier and cell membrane mimicry strategy confer excellent biocompatibility to PFB@CM. NIR laser irradiation provides sufficient tissue penetration for treating superficial tumors. Furthermore, the multimodal synergy allows therapeutic flexibility, as NIR parameters can be adjusted based on tumor characteristics to optimize the balance among different modalities.

Despite its promising antitumor efficacy, PFB@CM faces challenges for clinical translation. First, both PTT and BNN6 activation depend on 808-nm laser irradiation, with a tissue penetration depth of 1 to 2 cm, limiting application to superficial tumors. Future research should explore triggering methods with deeper penetration, such as NIR-II lasers or magnetothermal effects, for treating deep-seated or large tumors. Second, while a cascade reaction between NO and ROS was observed, the spatiotemporal control and optimal generation ratio require further elucidation. Since low NO concentrations can promote tumor growth, precise regulation of NO and ROS release is essential to maximize efficacy and minimize risks. Third, preliminary in vivo tests indicated no acute toxicity or organ damage, but comprehensive assessments of long-term safety, biodistribution, and metabolic pathways are necessary to ensure clinical safety. Finally, although MCF-7 cell membrane encapsulation validated homologous targeting, clinical adoption might necessitate personalized approaches using patient-derived cancer cell membranes for more precise targeting.

## Conclusion

In conclusion, we successfully developed a biomimetic nanomotor, PFB@CM, which utilizes homologous targeting for targeted delivery to tumor sites and enables multimodal synergistic therapy through rational material design and functional integration. Under NIR laser irradiation, PFB@CM exhibited excellent photothermal properties and self-thermophoretic motion. The incorporated Fe(II) engages in Fenton-like reactions with H_2_O_2_ within the tumor microenvironment to generate toxic ROS, facilitating CDT. NIR irradiation not only accelerates Fe(II) release to enhance CDT efficacy but also decomposes the thermally sensitive NO donor BNN6, enabling spatiotemporally controlled NO release. The ROS and NO generated subsequently undergo cascade reactions to form more active RNS, substantially augmenting tumor eradication. Both in vitro and in vivo evidence confirmed that PFB@CM effectively inhibits tumor growth via synergistic PTT and RNS/ROS therapy, while exhibiting excellent biocompatibility without causing significant histopathological damage to major organs. This study establishes an intratumoral NO controllable release system and NO-ROS cascade reaction pathway for generating RNS, thereby providing novel designs for the application of light-driven nanomotors in targeted cancer therapy.

## Data Availability

The data are freely available upon request.
