# Supplementary material for: NIR-Propelled Biomimetic Nanomotors for Photothermal/Chemodynamic/NO Synergistic Tumor Therapy
Source: Cyborg Bionic Syst. 2026 Apr 24;7:0495. doi: 10.34133/cbsystems.0495 (PMC13106939; doi:10.34133/cbsystems.0495)
Supplement: Supplementary 1 — Figs. S1 to S6 Movie S1 [file cbsystems.0495.f1.zip › Supporting Information.docx]

Supporting Information

**NIR-Propelled** **Biomimetic Nanomotors for Photothermal/Chemodynamic/NO Synergistic Tumor Therapy**

Ming Yang^1,2^, Jian Hu^2^, Zerui Li^2^, Hanhan Xie^3^, Hongri Gu^4^, and Chengzhi Hu^2^*

^1^School of Materials Science and Engineering, Harbin Institute of Technology, Harbin 150001, China

^2^Shenzhen Key Laboratory of Biomimetic Robotics and Intelligent Systems, Department of Mechanical and Energy Engineering, Southern University of Science and Technology, Shenzhen, 518055, China

^3^School of Optoelectronic Engineering, Guangdong Polytechnic Normal University, Guangzhou 510665, China

^4^Division of integrative Systems and design, Hong Kong University of Science and technology, Hong Kong, China.

^*^Address correspondence to: hucz@sustech.edu.cn

**Materials and methods**

**Equipment**

The morphology of the nanoparticles was characterized using scanning electron microscopy (SEM, Apreo2 S Lovac, FEI, Czech). The structural features and elemental distribution were characterized using transmission electron microscopy (TEM, Talos F200X G2, FEI). Zeta potentials were measured using a ZetaPALS instrument (BI-200SM, Brookhaven, USA). UV-vis-NIR absorbance spectra were recorded using a UV-visible spectrophotometer (Lambda 750s, PerkinElmer).

**Synthesis of BNN6**

BNN6 was synthesized following previously reported procedures. Briefly, 2.34 mL of N,N′-bis-sec-butylamino-p-phenylenediamine (BPA, 10 mmol) was dissolved in 18 mL of anhydrous ethanol. 20 mL of degassed sodium nitrite (6 M) was added under N2 protection, and the mixture was stirred for 30 min. Subsequently, 20 mL of hydrochloric acid solution (6 M) was added dropwise using a separatory funnel. The reaction was continued for 4 h, after which the BNN6 product was collected by centrifugation, washed with 50% (v/v) aqueous ethanol and lyophilized to obtain the final solid.

**Preparation of MCF-7 cell membranes**

Cell membranes were extracted from cultured MCF-7 cells using a combination of freeze-thaw cycles and differential centrifugation. To minimize protein degradation and preserve biological activity, all procedures were performed on ice or at 4°C. The extraction protocol was carried out as follows: Upon reaching >90% confluence, MCF-7 cells were harvested with a cell scraper (approximately 3×10⁷ cells per tube) and washed with ice-cold PBS. After removing residual PBS, the cell pellet was resuspended in Membrane Protein Extraction Reagent A supplemented with 1 mM phenylmethylsulfonyl fluoride (PMSF) and incubated on ice for 15 minutes. Cell disruption was achieved through three cycles of freezing in liquid nitrogen followed by thawing in a room-temperature water bath. The homogenate was then centrifuged at 700 g for 10 min at 4°C to remove nuclei and unbroken cells. The resulting supernatant was carefully collected and centrifuged at 14,000 g for 30 min at 4°C. The final cell membrane pellet was stored at −80°C for subsequent use.

**Photothermal properties of PFB**

PFB dispersions at various concentrations (0, 50, 100, 150, and 200 ppm) were irradiated with an 808 nm laser at a power density of 1.0 W/cm2 for 600 s. To investigate the effect of power density on photothermal properties, the PFB dispersions were irradiated with 808 nm lasers of different power densities (0.5, 1.0, 1.5, and 2.0) for 600 s. Real-time temperature changes were monitored throughout the irradiation process using an infrared thermographic camera.

**Cell cultures**

The MCF-7 cells were obtained from the China-type culture collection (CTCC) and cultured in high-glucose DMEM media (culture medium contains 10% FBS and 1% penicillin/streptomycin). Human umbilical vein endothelial cells (HUVEC) were purchased from Guangzhou CELLCOOK Biotech and cultured in low-glucose DMEM media (culture medium contains 10% FBS and 1% penicillin/streptomycin). The cells were cultured at 37°C incubator with 5% CO_2_.

**Cytotoxicity evaluation**

The in vitro toxicity of PFB@CM to MCF-7 cells was evaluated by CCK-8 assay. Briefly, cells were inoculated into 96-well plates at a density of 1 × 10^4^ cells per well. After 24 h of incubation, the original medium was replaced with 200 µL of fresh medium containing different concentrations of PFB@CM (0, 25, 50, 100, and 200 ppm). After 24 hours of incubation, the medium was replaced with 10% CCK-8 medium and incubated for another 60 min. Cell viability was measured by measuring absorbance at 450 nm using a microplate reader.

**Cellular uptake of PFB@CM.**

DOX-labeled PFB@CM was first prepared by incubating a mixture of PFB particles and MCF-7 cancer cell membranes with DOX (1 mg/mL) on an air shaker at low temperature for 24 h. After centrifugation and washing, the resulting DOX-PFB@CM was redispersed in cell culture medium for subsequent experiments. MCF7 and HUVEC cells were seeded in 48-well plates at a density of 1 × 10^5^ cells per well and cultured overnight. The medium was then replaced with fresh medium containing DOX-labelled PFB@CM. Following 4 hours of incubation, the cells were washed three times with PBS. Nuclei were stained with Hoechst-33342 and visualized using an inverted fluorescence microscope.

**Supplementary figures**

**
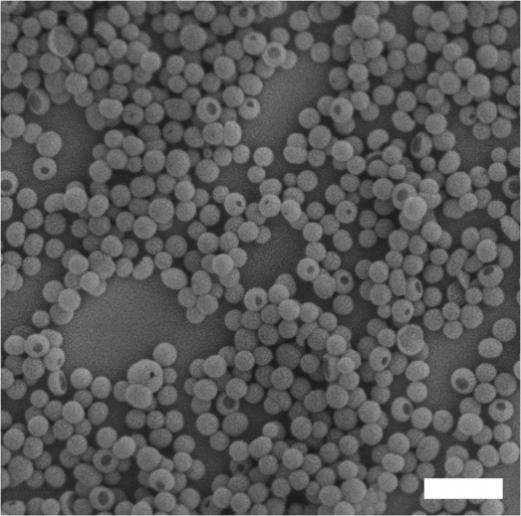
**

**Figure S1.** SEM image of PFB nanoparticles. Scale bar: 1 μm.





**Figure S2.** UV-vis absorption spectra of MB degradation under different experimental conditions.





**Figure S3.** Time-dependent absorbance changes of MB during degradation in the PFB + H_2_O_2_ group.


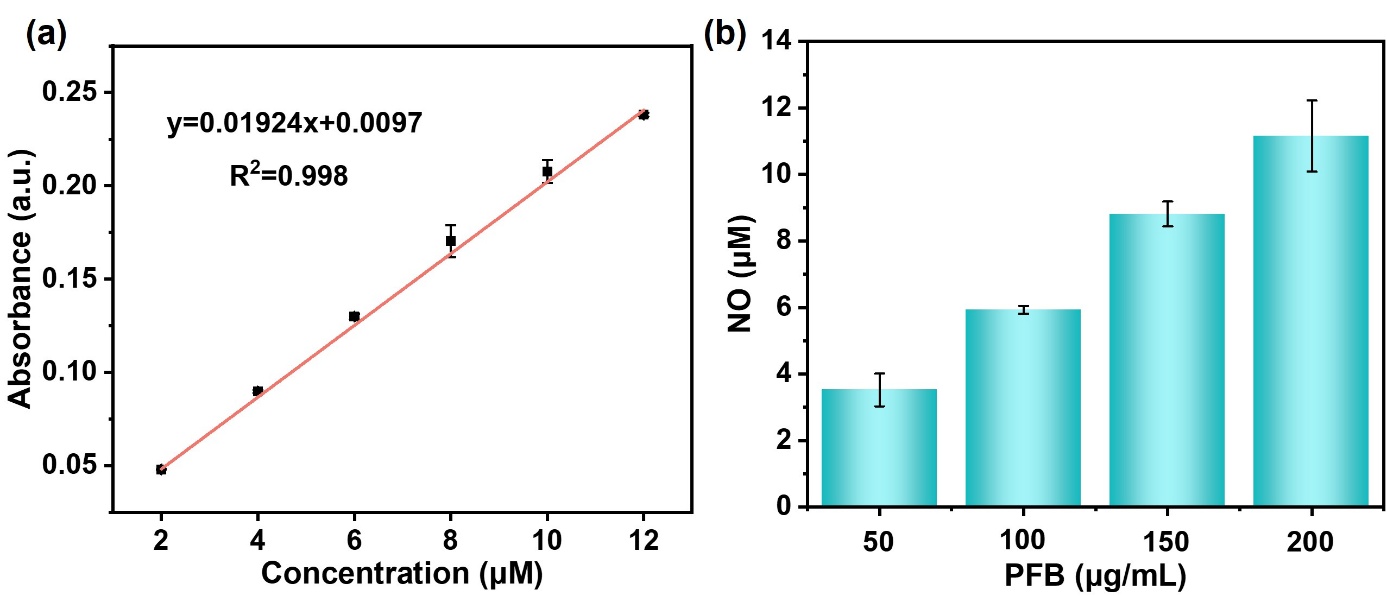


**Figure S4.** (a) Standard calibration curve for NO quantified using the Griess reagent. (b) Concentration-dependent NO generation from PFB under NIR irradiation.





**Figure S5.** Cell viability of MCF-7 and HUVEC cells after treatment with varying concentrations of PFB@CM


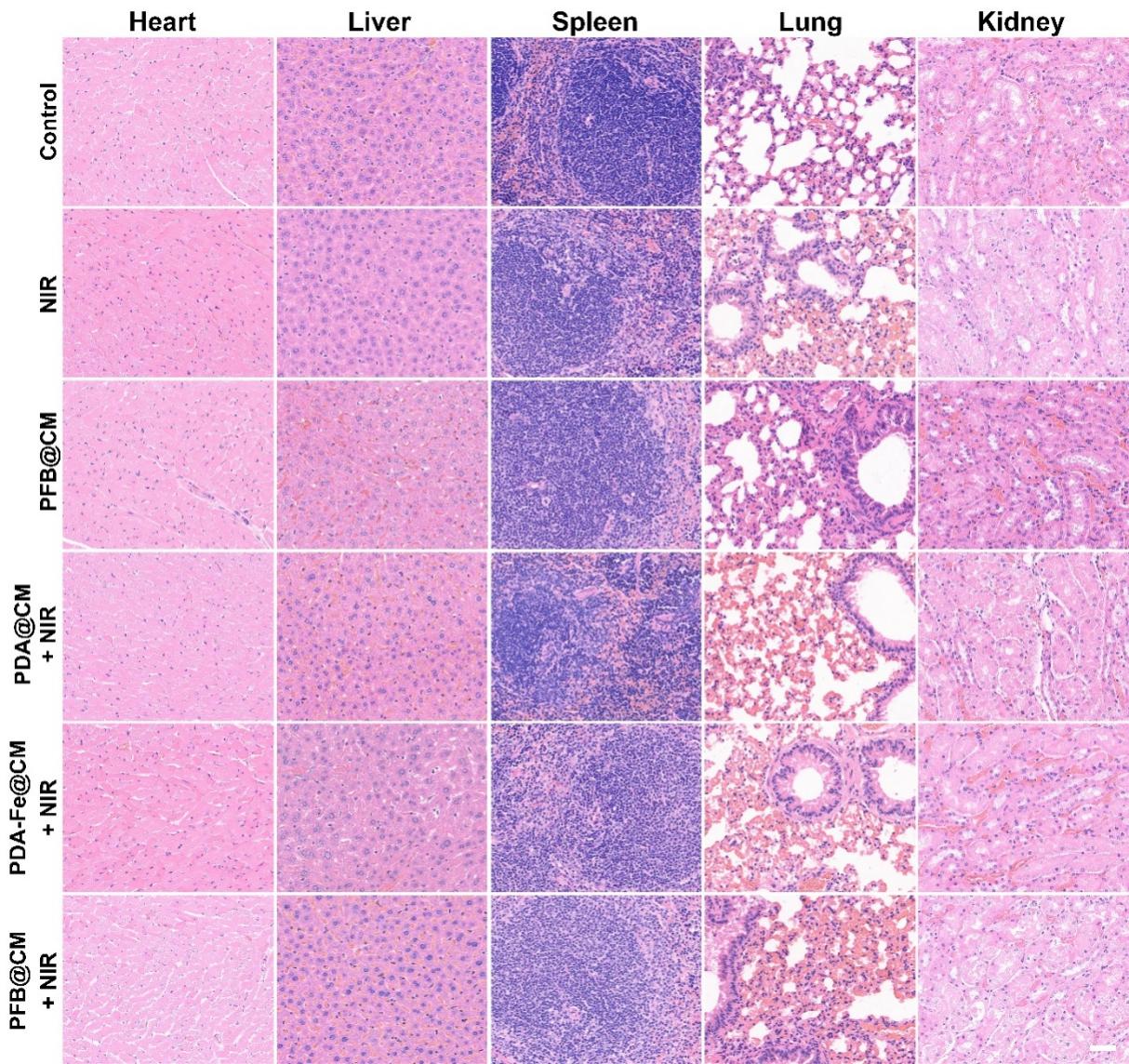


**Figure S6.** Representative H&E stained images of major organs (including heart, liver, spleen, lungs, and kidneys) from mice in different treatment groups after 14 days. Scale bar: 50 μm.
